# 1849. Impact of Medicaid Expansion on Human Immunodeficiency Virus Outcomes in Nebraska

**DOI:** 10.1093/ofid/ofad500.1677

**Published:** 2023-11-27

**Authors:** James M McCluskey, Renae Furl, Monica Arroyo, Nada Fadul

**Affiliations:** University of Nebraska Medical Center, Omaha, Nebraska; University of Nebraska Medical Center, Omaha, Nebraska; University of Nebraska Medical Center, Omaha, Nebraska; University of Nebraska Medical Center, Omaha, Nebraska

## Abstract

**Background:**

Human immunodeficiency virus (HIV) viral suppression (VS) is a major predictor for improved clinical outcomes and decreased viral transmission in patients with HIV (PWH). There is evidence to suggest that enrollment in the Affordable Care Act (ACA) qualified health plan is associated with improved VS, however there is inconsistency in the literature on whether Medicaid enrollment can lead to similar outcomes. Previous research has shown that PWH living in Medicaid expansion states are more likely to be covered by Medicaid and less likely to be uninsured compared to those who live in non-expansion states. In 2014, the ACA Medicaid expansion went into effect and expanded Medicaid coverage to most adults with an income up to 138% of the Federal Poverty Level (PFL), which is $14,580 for an individual in 2023. Nebraska implemented the expansion October 2020. This provided an opportunity to further evaluate this gap in the literature using the data from the main provider of HIV care in Nebraska, the University of Nebraska Medical Center Specialty Care Center (UNMC SCC).

**Figure:**
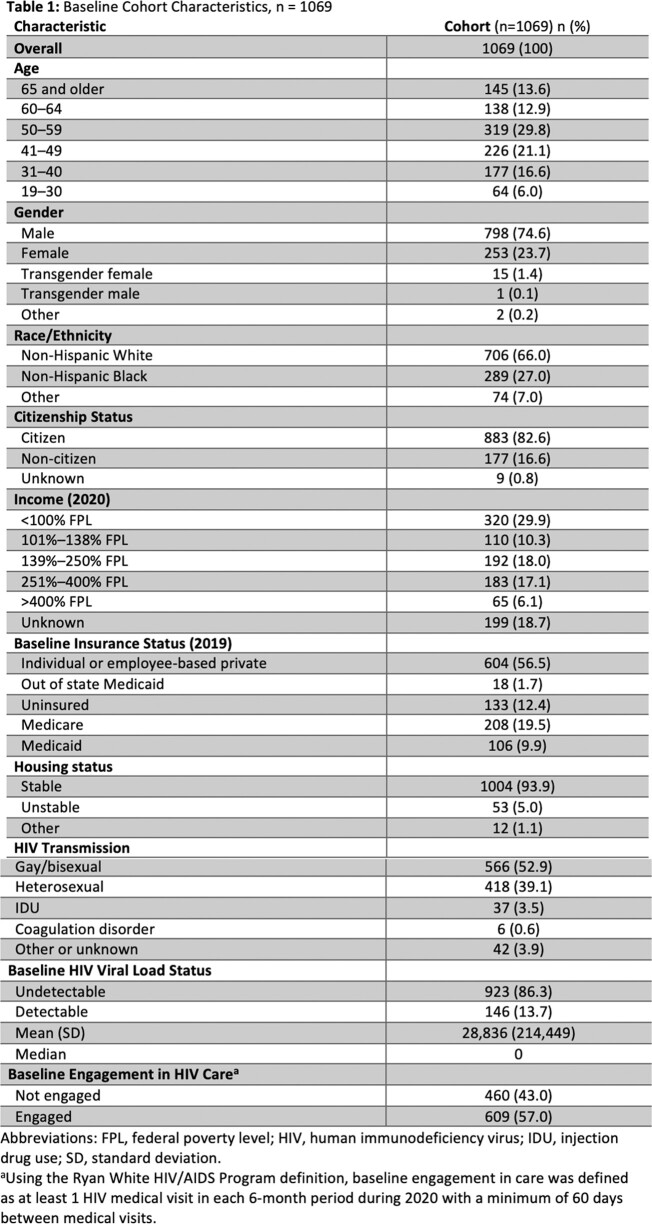

**Figure:**
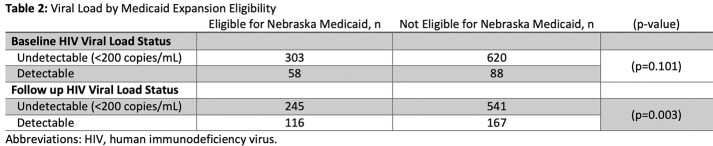

**Methods:**

PWH were recruited from UNMC SCC for a retrospective observational study that included all patients 19 years of age or older that had at least one HIV provider clinic visit in the preceding 12 months before October 2020. If the patients had unknown income or were below 138% FPL and were not already enrolled in Medicare or out of state Medicaid, they were considered eligible for Medicaid expansion. PWH that were eligible and those that were not eligible for Medicaid expansion then had follow up viral loads compared to assess for VS.

**Results:**

A diverse group of 1,069 patients were included in the study (Table 1). No statistically significant result was found between baseline VS, however those that were not eligible for Medicaid expansion showed a greater level of VS at follow up (Table 2), p=0.003.

**Conclusion:**

This retrospective study found that approximately 1 year after Medicaid expansion in Nebraska, PWH that were not eligible for Medicaid expansion had better VS. This could be explained by a lack of those that were newly eligible for Medicaid being able to enroll among other reasons. This finding warrants further evaluation to assess for barriers to care, including but not limited to, Medicaid enrollment and its contribution to HIV VS.

**Disclosures:**

**All Authors**: No reported disclosures

